# Supplementary material for: Up for the challenge: Power motive congruence drives nurses to craft their jobs and experience well-being
Source: PLoS One. 2024 Oct 3;19(10):e0310717. doi: 10.1371/journal.pone.0310717 (PMC11449283; doi:10.1371/journal.pone.0310717)
Supplement: S1 File — (DOCX) [file pone.0310717.s001.docx]

**Supporting Information**

**S1.a. Development and Validation of the Adapted Job Crafting Scale**

***General Description***

In an aim to make job crafting items reflect rather implicit motivation behind their demonstration, we used the coding categories of the Operant Multi-Motive Test (OMT; Kuhl, 2001). The OMT is a Picture Story Exercise (PSE) (Schultheiss, 2008) that originates from the Thematic Apperception Test (TAT; Murray, 1943). Similar to the basic concept of the TAT, the OMT is a projective measure that asks participants to respond to open-ended questions about pictures. Answers to the OMT are usually coded based on their motive contents using the OMT Manual (Kuhl & Scheffer, 1999) that includes the five motive enactment categories for each of the big three motives (Achievement, Affiliation, and Power). We chose to adapt the validated and widely used scale developed by Tims et al. (2012). The scale includes 21 items assessing the four dimensions of job crafting: 1) increasing structural job resources; 2) increasing social job resources; 3) increasing challenging job demands; and 4) decreasing hindering job demands.

As a first step, we decided to group the items into those that describe employees *approaching* a certain situation, and others that describe employees *avoiding* a certain situation (see also Bipp & Demerouti, 2015). We decided to perform such a grouping since the promotion focus was found to positively relate to the “seeking resources and challenges” dimensions of job crafting, while the prevention focus relates to its “reducing demands” dimension (Petrou & Demerouti, 2015). Moreover, Bipp and Demerouti (2015) explored the relationship between basic personality dimensions and job crafting and supported that scoring high on approach temperament positively related to seeking resources and demands, while scoring high on avoidance temperament positively related to reducing hindering demands at work.

For each of the items that were present in the original job crafting scale, we decided to develop one item that is more associated with explicit motives and one that might be more related to implicit motives.

Generally, implicit motives function as a network that associates situational prompts with basic affective reactions and implicit behaviors (McClelland et al., 1953). For that reason, developing items that better relate to implicit motives requires adding an affective component and a hedonic element to the item that go beyond the apparent instrumental value of the activity. For the examples above, employees can indicate that they develop their capabilities because they enjoy the actual process of self-development irrespective of the possibility of receiving a reward or being recognized. The above-mentioned instrumentality is specified in the adaptation of the items that are geared more towards the explicit motivational system, which is strongly influenced by social norms and interactions (Koestner et al., 1991; McClelland, 1985). We added, for instance, an anticipation of a reward such as getting a promotion or attaining a desirable position at work. The same method guided us through the process of developing all the other items.

The table below outlines the items that belong to the job crafting dimension of increasing challenging job demands developed by Tims et al. (2012) and the corresponding items that were adapted in a way to either highlight an instrumental or an affective orientation. Only the adapted items were used in the study.

| *Original and Adapted Job Crafting Items and their Corresponding Orientations* | | |
| --- | --- | --- |
| **Original Item** | **Adapted Item** | **Orientation** |
| When an interesting project comes along, I offer myself proactively as project co-worker | When an interesting project comes along, I proactively offer myself to enhance my portfolio/ resume | Instrumental |
|  | I enjoy proactively offering myself when an interesting project comes along | Affective |
| If there are new developments, I am one of the first to learn about them and try them out | I am always the first one to try new developments out to enhance my skills | Instrumental |
|  | I enjoy challenging myself by trying new developments out | Affective |
| When there is not much to do at work, I see it as a chance to start new projects | When there is not much to do at work, I see it as a chance to start new projects to enhance my abilities | Instrumental |
|  | I am always eager to start new projects when there is not much to do at work because I dislike feeling idle | Affective |
| I regularly take on extra tasks even though I do not receive extra salary for them | I take on extra tasks because this is part of my performance appraisal | Instrumental |
|  | I enjoy taking on extra tasks at work | Affective |
| I try to make my work more challenging by examining the underlying relationships between aspects of my job | I try to understand how my work tasks are related to one another | Instrumental |
|  | I enjoy finding out how my tasks at work are related to one another | Affective |

***Scale Piloting Study***

*Aim.* This study aimed to adapt, extend, and establish the internal and external validity of a job crafting scale that targets the implicit side of job crafting using an international sample of workers.

*Sample.* After securing ethical approval (OSB.LD.21 and EC-2017.EX99) from the institutions of the authors, we collected data from a pool of American workers via Amazon Mechanical Turk (MTurk). A second sample was a convenience sample of employees who were invited by the authors to participate through a link to the electronic survey. Principal components / exploratory factor analysis requires the presence of 10 participants per item (Tabachnick & Fidell, 2007). The adapted scale is made up of 42 items which means that the minimum number that we should aim for is at least 420 (42*10) participants per sample.

*Measures.* The survey included a demographics section followed by the adapted job crafting scale. The 6-item version of the Proactive Personality Scale (PPS; Bateman & Crant, 1993) that was developed by Claes et al. (2005), the 7-item Personal Initiative Scale (Frese et al., 1997), and the 8-item cynicism scale developed by Wanous et al. (2000).

*Analysis and results.* The reliability scores of the sub-dimensions were all above .80 when combined (affective and instrumental). EFA and CFA indicated a better fit with w four-dimensional model instead of an 8-dimensional one. Moreover, the affective and instrumental versions of the job crafting scale correlated highly with one another (e.g. the correlation between affective increasing challenging job demands and instrumental increasing challenging job demands was .84**). Finally, convergent and divergent validity tests showed that the sub-dimensions of increasing challenging job demands, increasing social job resources, and increasing structural job resources correlated positively with proactive personality (*r* =.65**, *r* = .47**, and *r* = .70** respectively) and with personal initiative (*r* =.71**, *r* = .45**, and *r* = .70** respectively) while correlating negatively with cynicism (*r* = - .29**, *r* = - .18**, and *r* = - .44** respectively). However, decreasing hindering demands was negatively correlated with proactive personality (*r* = - .13**) and with personal initiative (*r* = - .23**), while positively correlated with cynicism (*r* = .36**).

References

Bateman, T. S., & Crant, J. M. (1993). The proactive component of organizational behavior: A measure and correlates. *Journal of Organizational Behavior*, *14*(2), 103-118.

Bipp, T., & Demerouti, E. (2015). Which employees craft their jobs and how? Basic dimensions of personality and employees' job crafting behaviour. *Journal of Occupational and Organizational Psychology*, *88*(4), 631-655.

Claes, R., Beheydt, C., & Lemmens, B. (2005). Unidimensionality of abbreviated proactive personality scales across cultures. *Applied Psychology*, *54*(4), 476-489.

Frese, M., Fay, D., Hilburger, T., Leng, K., & Tag, A. (1997). The concept of personal initiative: Operationalization, reliability and validity in two German samples. *Journal of Occupational and Organizational Psychology*, *70*(2), 139-161.

Koestner, R., Zuroff, D. C., & Powers, T. A. (1991). Family origins of adolescent self-criticism and its continuity into adulthood. *Journal of Abnormal Psychology*, *100*(2), 191.

Kuhl, J. (2001). *Motivation und persönlichkeit: Interaktionen psychischer systeme. [Motivation and Personality: Interactions of Mental Systems].* Hogrefe.

Kuhl, J., & Scheffer, D. (1999). Der operante multi-motiv-test (OMT): Manual [The operant multi-motive-test (OMT): Manual]. *Germany: University of Osnabrück*.

McClelland, D. C. (1985). How motives, skills, and values determine what people do. *American Psychologist*, *40*(7), 812.

McClelland, D. C., Atkinson, J. W., Clark, R. A., & Lowell, E. L. (1953). *The Achievement Motive*. Appleton-Century-Crofts.

Murray, H. A. (1943). *Thematic apperception test manual*. Harvard University Press.

Petrou, P., & Demerouti, E. (2015). Trait-level and week-level regulatory focus as a motivation to craft a job. *Career Development International*, *20*(2), 102-118.

Schultheiss, O. C. (2008). Implicit motives. In O. P. John, R. W. Robins, & L. A. Pervin (Eds.), *Handbook of personality: Theory and research* (3rd ed., pp. 603–633). Guilford.

Tabachnick, B. G., & Fidell, L. S. (2007). *Experimental designs using ANOVA* (Vol. 724). Thomson/Brooks/Cole Belmont, CA.

Tims, M., Bakker, A. B., & Derks, D. (2012). Development and validation of the job crafting scale. *Journal of Vocational Behavior*, *80*(1), 173-186.

Wanous, J. P., Reichers, A. E., & Austin, J. T. (2000). Cynicism about organizational change: Measurement, antecedents, and correlates. *Group & organization management*, *25*(2), 132-153.
