# Supplementary material for: Up for the challenge: Power motive congruence drives nurses to craft their jobs and experience well-being
Source: PLoS One. 2024 Oct 3;19(10):e0310717. doi: 10.1371/journal.pone.0310717 (PMC11449283; doi:10.1371/journal.pone.0310717)
Supplement: S2 File — (DOCX) [file pone.0310717.s002.docx]

**S1.b. Coding the OMT**

The OMT coding procedure starts by checking for the presence of motive imagery (affiliation, achievement, power), otherwise, a ‘‘zero’’ is coded, indicating no motive imagery. After determining the motive content, the coder determines the realization of this motive according to the five enactment strategies (Kuhl & Scheffer, 1999). If approach behavior is present, then the coder selects one of the levels 1 to 4, if avoidance behavior is present, the coder uses level 5. This last level is a combination of negative affect and passivity. The next step is to check for the affect that is guiding this behavior. Levels one and two are guided by positive affect, with level one being intrinsically driven and self-regulated, and the second level being more incentive driven. The third and fourth levels are guided by negative affect, with the third level describing a more self-regulatory way of coping with negative emotions, and the fourth level an incentive-driven way to reduce negative affect (Baumann et al., 2010; Kuhl & Scheffer, 1999; Kuhl et al., 2003). The fifth level describes being overpowered by negative affect like fear, guilt, or loneliness that hinders self-regulatory abilities (Kuhl, 2001). For the purpose of our investigation, we focused on the third level of the power motivation in the OMT. Coding the answers was conducted by the first author who is bilingual (English, Arabic), and underwent coding training with the second author until the percentage of agreement between the trainer and the trainee reached more than 80%.

References

Baumann, N., Kazén, M., & Kuhl, J. (2010). Implicit motives: A look from personality systems interaction theory. In O. C. Schultheiss & J. C. Brunstein (Eds.), *Implicit Motives* (pp. 375-403). Oxford University Press.

Kuhl, J. (2001). *Motivation und persönlichkeit: Interaktionen psychischer systeme. [Motivation and Personality: Interactions of Mental Systems].* Hogrefe.

Kuhl, J., & Scheffer, D. (1999). Der operante multi-motiv-test (OMT): Manual [The operant multi-motive-test (OMT): Manual]. *Germany: University of Osnabrück*.

Kuhl, J., Scheffer, D., & Eichstaedt, J. (2003). Der Operante Motiv-Test (OMT): Ein neuer Ansatz zur Messung impliziter Motive. [The Operant Motive Test (OMT): A New Approach to Measuring Implicit Motives]. *Diagnostik von motivation und selbstkonzept*, 129-149.
