# Supplementary material for: Up for the challenge: Power motive congruence drives nurses to craft their jobs and experience well-being
Source: PLoS One. 2024 Oct 3;19(10):e0310717. doi: 10.1371/journal.pone.0310717 (PMC11449283; doi:10.1371/journal.pone.0310717)
Supplement: S3 File — (DOCX) [file pone.0310717.s003.docx]

**S1.c. Interpretations of Low Reliability of the Integrative Explicit Power Motivation Scale**

First, we collected data from a sample of nurses from a non-WEIRD (Western, Educated, Industrialized, Rich, and Democratic) population (Henrich et al., 2010) using scales that were developed in a Western context, which might have influenced the psychometric properties of the scales. Many scales, such as this one, have been developed without ensuring that the constructs that were created in WEIRD populations are applicable in non-WEIRD contexts. Individuals from different cultural backgrounds have different experiences and assumptions that might cause them to interpret words and phrases, scale items, or sometimes the entire scale differently (He & van de Vijver, 2012; Leung & Van De Vijver, 2008; van de Vijver & Leung, 1997, 2021). The alpha reported by the original developers of the scale was satisfactory (α=0.70) (Kuhl, 1999) , however, it is at the lower limit of the acceptable range of internal consistency scores, therefore making its use in a non-WEIRD sample more threatening to its internal consistency. The second reason is related to problems with the conditions that need to be satisfied for Cronbach’s alpha to be an accurate estimation of reliability. One of those conditions is unidimensionality, which is when the item responses are independent after controlling for a single latent factor (Reise et al., 2007) or when all the items load unto a single factor in a factor analysis. The problem with this condition is that in social sciences, item response matrices are rarely perfectly unidimensional (Reise et al., 2007). The complexity of the constructs being measured makes it necessary to enrich the content of the items being used to better capture the construct (Reise et al., 2007; Teo & Fan, 2013), which might influence the unidimensionality of the scale. For example, the developers of the Ten Item Personality Inventory (TIPI; Gosling et al., 2003) clarify that they sought to maximize content validity and breadth of coverage, while avoiding redundancy. Thus, it is not surprising that the TIPI has an internal consistency lower that other similar tools (Romero et al., 2012). The third reason is that the low reliability score might be related to the length of the scale as it is a short four-item scale. Kline (2013) has questioned the use of the alpha coefficient for very short scales and some authors have suggested that low reliability scores are expected in short scales (Sprecher et al., 1994). In extreme cases, such as single-item scales, traditional tools that rely on internal consistency cannot be adopted and are therefore not presented (Cheung & Lucas, 2014). Cronbach’s can be a problematic estimate of reliability for short scales, making their reliabilities lower (e.g., test– retest, internal consistency) than long scales, but still satisfactory (Ziegler et al., 2014).

References

Cheung, F., & Lucas, R. E. (2014). Assessing the validity of single-item life satisfaction measures: Results from three large samples. *Quality of Life Research*, *23*(10), 2809-2818.

Gosling, S. D., Rentfrow, P. J., & Swann Jr, W. B. (2003). A very brief measure of the Big-Five personality domains. *Journal of Research in Personality*, *37*(6), 504-528.

He, J., & van de Vijver, F. (2012). Bias and equivalence in cross-cultural research. *Online Readings in Psychology and Culture*, *2*(2), 2307-0919.1111.

Henrich, J., Heine, S. J., & Norenzayan, A. (2010). The weirdest people in the world? *Behavioral and brain sciences*, *33*(2-3), 61-83.

Kline, P. (2013). *Personality: The psychometric view*. Routledge.

Kuhl, J. (1999). Der Motiv-Umsetzungs-Test (MUT)[The motive-enactment-test (MUT)]. *Unpublished questionnaire, University of Osnabrück, Osnabrück, Germany*.

Leung, K., & Van De Vijver, F. J. (2008). Strategies for strengthening causal inferences in cross cultural research: The consilience approach. *International Journal of Cross Cultural Management*, *8*(2), 145-169.

Reise, S. P., Morizot, J., & Hays, R. D. (2007). The role of the bifactor model in resolving dimensionality issues in health outcomes measures. *Quality of Life Research*, *16*(1), 19-31.

Romero, E., Villar, P., Gómez-Fraguela, J. A., & López-Romero, L. (2012). Measuring personality traits with ultra-short scales: A study of the Ten Item Personality Inventory (TIPI) in a Spanish sample. *Personality and Individual Differences*, *53*(3), 289-293.

Sprecher, S., Aron, A., Hatfield, E., Cortese, A., Potapova, E., & Levitskaya, A. (1994). Love: American style, Russian style, and Japanese style. *Personal Relationships*, *1*(4), 349-369.

Teo, T., & Fan, X. (2013). Coefficient alpha and beyond: Issues and alternatives for educational research. *The Asia-Pacific Education Researcher*, *22*(2), 209-213.

van de Vijver, F. J., & Leung, K. (1997). *Methods and data analysis for cross-cultural research*. Sage.

Van de Vijver, F. J., & Leung, K. (2021). *Methods and data analysis for cross-cultural research*. Cambridge University Press. <https://doi.org/https://doi.org/10.1017/9781107415188>

Ziegler, M., Kemper, C. J., & Kruyen, P. (2014). Short scales–Five misunderstandings and ways to overcome them. In: Hogrefe Publishing.
