# Supplementary material for: Up for the challenge: Power motive congruence drives nurses to craft their jobs and experience well-being
Source: PLoS One. 2024 Oct 3;19(10):e0310717. doi: 10.1371/journal.pone.0310717 (PMC11449283; doi:10.1371/journal.pone.0310717)
Supplement: Attachment — Submitted filename: Response to Reviewers.docx [file pone.0310717.s004.docx]

| Dear Editor, dear reviewers,  We would like to thank you for the valuable feedback that you have provided us with. We believe that this considerably improved our paper, and we hope that the changes that we made fully address your comments.  Below, we have delineated your comments, provided our corresponding responses, and indicated the locations where revisions have been implemented within the paper. We also highlighted the changes that we made in yellow in the revised manuscript.  We really appreciate the time that you put into revising our paper.  Best regards,  Rawan Ghazzawi on behalf of the co-authors | | | |
| --- | --- | --- | --- |
| **Code** | **Reviewer’s Comment** | **Our Response** | **Pages/Lines Changes Made** |
|  | **Title** |  |  |
| R2-1 | Title: it does not fully inform about the sample characteristics and the relationships tested in the paper. Please, try to better address this point. | Thank you for your comment. We agree with you and we changed the title accordingly. | Pages 1&3 |
|  | **Abstract** |  |  |
| R1-1 | I think the abstract can be shortened and make it more accesible. | We agree with you and we shortened the abstract accordingly. | Page 2 |
| R1-2 | How are needs and self-attributed needs any different? | We added this sentence to the abstract : “While implicit motives tend to operate at the unconscious, explicit motives operate at the unconscious level.” | Page 2 |
| R1-3 | In the abstract you report that “Power motives are characterized by the need to have an impact on others” but job crafting is mainly related to the self, hence it is unclear how these motives fit the overall idea of jc. | We do agree with you that JC is mostly about the self, which is why we are trying to link it to implicit and explicit basic needs. However, our focus here is about the “how” and choosing the power motivation was driven by the idea that we seek to influence our surroundings (prosocially or in a suppressive manner) in order to satisfy our personal need for power, so it is still about the self. But we agree with you that the way we portrayed it might be misleading.  Accordingly, we updated the sentence and now it is : “We focused on power motives, as power is an agentic motive characterized by the need to influence your environment.” | Page 2, lines 12-13 |
| R1-4 | It is unclear how H4 fits with the overall research question of the study. I do not think you need to specify all the hypotheses in the abstract. | We removed the hypotheses from the first part of the abstract. When it comes to H4, we added it in order to try to justify as much of the relationships as possible in the study. The two outcomes that we have chosen ( job satisfaction and work engagement) are very relevant to the nursing profession, JC and to each other and it would have made the model weaker if we had not accounted for the relations between them. Although this relationship is not directly related to the RQ, it helps us understand the impact that JC might have on the nurses who engage in it. | No change |
| R1-5 | The study design should be described better in the abstract. | We considered your comment and we added more information to the abstract. | Page 2 |
| R1-6 | You refer to integrative power motive enactment, which is rather unclear in the abstract. Also, from the abstract, it is unclear why you only focused on seeking challenges. | We hope that the changes that we made in the abstract make it clearer now.  When it comes to your comment about why we focused on increasing challenging job demands, we added this sentence that we hope clarifies this:  “Although power is relevant to job crafting in its entirety, in this study, we link it to increasing challenging job demands due to its relevance to job control, which falls under the umbrella of power.” | Page 2, lines 13-15 |
| R1-7 | What do you mean by “integrative” power? The use of different terms and acronyms makes it more difficult to follow your paper. | We mention the word integrative to indicate the level of power we are addressing. At this level, individuals can integrate their emotions (especially negative ones) in a mature manner allowing them to recover after experiencing them and even grow. | No change |
| R1-8 | Why should increasing challenges be “one of the most important dimensions of job crafting”? | Thank you for your comment. We base this idea on the results of the Meta-analytic confirmatory factor analysis conducted by Rudolph et al (2017), which shows that increasing challenging job demands has a highest loading unto overall JC (.811*) and explaining the largest variance (R2 = .657). | No change |
|  | Introduction |  |  |
| R1-9 | You report that research on job crafting has so far ignored basic motives for power, affiliation and achievement. I do not think this is correct, and I encourage the authors to carefully review JC literature. For example, there are studies considering the link between social norms and job crafting, where social norms are related to the human need for affiliation. A more precise literature review is needed. | Thank you for mentioning this study. We agree with you that basic needs have already been explored and we are very aware of the literature on that such as:  Slemp, G. R., & Vella-Brodrick, D. A. (2014). Optimising employee mental health: The relationship between intrinsic need satisfaction, job crafting, and employee well-being. *Journal of Happiness Studies*, *15*, 957-977.  Bipp, T., & Demerouti, E. (2015). Which employees craft their jobs and how? Basic dimensions of personality and employees' job crafting behaviour. *Journal of occupational and organizational psychology*, *88*(4), 631-655.  However, these studies focus only on explicit needs which can only be measured via projective measures and not on implicit needs. Having mentioned that, we thank you for pointing this out to us and for providing us with additional information. Accordingly, we have updated the sentence:  “Studies on personal antecedents of job crafting have mainly focused on traits such as the big five and self-efficacy (3) and basic needs at the explicit (intrinsic and extrinsic) level (6, 7), which were restricted to self-report. However, implicit (unconscious) basic motives for power, achievement, and affiliation that are driving forces of human behavior (8, 9) have been neglected thus far” | Page 3, lines 40-44 |
| R1-10 | Line 55: you report correlation coefficients, but this is not needed here. | We have removed them. | Page 4, line 59 |
| R1-11 | Overall, the intro lacks convincing elaboration as to why this study was needed. There are many studies on the role of job crafting for work engagement and satisfaction, so merely stating that this one focuses on nurses is not enough, nor does it refer to the main overall research question of the study. I suggest the authors focus on their main research question and elaborate better as to why this is an important topic to investigate – adopting the research design you adopted – now. | We agree with you that the gap was not very clear in the introduction and accordingly, we added this section:  “however, we still do not know a lot about the personal antecedents of job crafting in general (4) and among nurses specific. This can create a level of unclarity as to who is more likely to engage in job crafting. Addressing this gap is essential as job crafting is a personal and proactive work behavior driven by basic needs (2, 5).” | Page 4, lines 34-37 |
| R1-12 | Line 74: I was not able to find the variance you report in Rudolph et al.’s paper. This being said I did not find it convincing your argument as to why focusing on seeking challenges only is appropriate, also given that research shows that the different job crafting dimensions function very differently. | Thank you for your comment. You can find this information on page 122 in  Rudolph, C. W., Katz, I. M., Lavigne, K. N., & Zacher, H. (2017). Job crafting: A meta-analysis of relationships with individual differences, job characteristics, and work outcomes. Journal of vocational behavior, 102, 112-138.  Although we partially agree with you, we also see the fact that a dimension explains that largest amount of variance in an overall construct is a substantial reason to focus on this dimension because it is also relevant as well to power motivation. Moreover, the fact that the JC dimensions seem to be very different can serve as more support for the need to focus on each JC dimension on its own rather that than on the entire construct of JC. | No change |
| R1-13 | On line 69 you report that job crafting consists of increasing and decreasing challenging job demands. I found this incorrect, as job crafting encompasses increasing challenging demands and decreasing hindering demands, plus optimizing demands, as for current research. Hindering and challenging demands are not the same and shouldn’t be treated as if they were. | Thank you for your comment. This was a typo and we have corrected it. | Page 4, lines 73-74 |
| R1-14 | Line 101: You report that nursing can be regarded as a power profession. I was not really convinced here about categorizing a profession as defined by a single motive. Could it be that there are different motives driving a profession?  Also because of this, I did not find it convincing that power motive is the most relevant driver of job crafting (line 106). | We agree with you that a single motive does not sum up an entire profession, however, research indicates that nurses chose nursing as a profession driven by a calling to care for and help others, which falls under the umbrella of the power motive. Of course, other motives like affiliation and achievement can also drive the profession, however, given the highly physically and emotionally demanding nature of the nursing profession and the less than ideal employment conditions offered, it is less likely that nurses are driven by the need for achievement and more by the need for power (helping).  However, we still think that we need to support our argument better and we thank you for pointing this out to us. Accordingly, we have indicated that :  “Although nurses are driven by different motives when making the decision to join the profession, most of them express that they chose this profession driven by a calling to help and care for others (36).” | Page 6, lines 107-109 |
| R1-15 | Line 127: You argue that job crafting starts at the cognitive level. However, some more recent research shows that job crafting behaviors seem to anticipate cognitive crafting (e.g., https://doi.org/10.1017/jmo.2022.79 ). Again, a more updated review is needed here to provide a stronger grounding to your hypotheses. | Thank you for your very helpful comment and for adding the doi! This indeed is a very useful and relevant study. Based on some of the results of this study and building on sensemaking theory, which is highlighted here :  *“Behaviors constitute the raw material of cognition, representing what is being made sense of, since people make sense of what they are doing while striving for coherence with their past behaviors (Melo, Dourado, & Andrade, Reference Melo, Dourado and Andrade2021; Weick et al., Reference Weick, Sutcliffe and Obstfeld2005). At the same time, people's cognitive processes are used as a reference to guide future actions, with people making sense of a situation which, in turn, drives following actions (Maitlis & Christianson, Reference Maitlis and Christianson2014).”*  We cannot invalidate the idea that JC starts at the cognitive level. Moreover, the results of the study indicated that :  “employees who reframed their work to highlight the relevance of its perceived resources later reported enriching their jobs and expanding their work roles more often, with benefits for their levels of work engagement over time.”  However, this was not the case for avoidance crafting, which is understandable. Additionally, we have evidence that shows that job crafting intentions are significantly related to actual job crafting (Tims et al., 2015), which further supports our claim that JC starts at the cognitive level.  Tims, M., Bakker, A. B., & Derks, D. (2015). Job crafting and job performance: A longitudinal study. *European Journal of Work and Organizational Psychology*, *24*(6), 914-928.  In order to address your comment, we have updated the section accordingly:  “Wrzesniewski and Dutton (5) argue that job crafting starts at the cognitive level and takes place in the workplace where performance indicators serve as expectations to be met. Zhang and Parker (44) have argued that cognitive crafting is relevant in driving job crafting behaviors. Moreover, a recent study by Costantini (45) indicted that employees who cognitively reframed their work engaged in more role expansion.” | Page 7, lines 134-138 |
| R1-16 | The extensive use of acronyms makes it really difficult to follow your arguments. Please revise using the same terms consistently and avoid acronyms as much as possible. | We have removed the acronyms in the paper and instead use only implicit power and explicit power. We hope that makes reading the paper clearer. | Across the entire paper |
| R2-2 | In the literature review section, you should revise the SPECIFIC research on job crafting among nurses. In the recent time, there is a lot of studies on job crafting with healthcare workers. Please, revise the relevant literature. | We agree with you that this was not very clear and accordingly we added the following information in the different sections:  “Moreover, a very limited number of these studies investigated the personal antecedents of job crafting in the nursing context (e.g., 17)”  “In the nursing context, this can be volunteering to help at the emergency department when there is a lot of pressure or creating a small support group for junior nurses.”  “Although nurses’ decisions to join the profession are driven by different motives, most of them express that they chose this profession driven by a calling to help and care for others (36).”  “Extensive research supports the relationship between job crafting and work engagement in general (e.g., 2, 27) and among nurses (57).” | Page 4, lines 53-54  Page 5, lines 83-84  Page 6, lines 107-109  Page 8, lines 162-163 |
| R2-3 | Some additional explanations about the relationships between job crafting and work engagement should be considered, both in the introduction and in the discussion. For instance, recent studies tested a longitudinal moderated mediation model in which Work Engagement increases over time through an increment in Job Crafting behaviors (Hypothesis 1), while this process is moderated by workers’ age (Hypothesis 2). Studies as Topa and Aranda, 2023 should be discussed as related to your findings. | Thank you for your comment and for including this very interesting study!  We have updated the section accordingly:  “According to the JD-R model, motivated employees engage in job crafting and as result have more resources and experience higher levels of motivation (58). This is referred to as the empirically “gain spiral” that employees create for themselves to increase their work engagement via job crafting (59). However, little is known about the motivational antecedent in this relationship. This is surprising, especially since work engagement is often referred to as a motivational process in the job demands-resources model (60).”  When it comes to the discussion, we have made the following changes as well:  “We extend this literature by supporting such a robust relationship in an underrepresented non-Western sample and by providing empirical support for the role of motives in increasing the level of motivation among employees (gain spiral) (58).” | Pages 8 & 9, lines 163-169  Page 18, lines 365-367 |
|  | **Method** |  |  |
| R1-17 | How did you recruit participants? What info was given regarding the study aims? In the intro, you referred to the pandemic, but the study was conducted before it, so that argument is not appropriate for the study. | In order to address your first two questions, we added the following information:  “Hardcopies of the questionnaires were distributed among the nurses in each of the participating hospitals and the nurses were requested to return the filled questionnaires. At the beginning of the study the participants had the chance to read about the aim of the study and provide their consent if applicable.”  When it comes to your last comment about the COVID-19 argument in the introduction, thank you and we agree with you. We have updated the argument accordingly:  “Knowing that the well-being of health care professionals is an important predictor of patient quality of care (18, 19) and individual and organizational performance (20, 21) it would be of high value to investigate the influence that job crafting has on important positive work outcomes in such a professional context.” | Page 10, lines 195-198  Page 4, lines 54-58 |
| R1-18 | Please detail the procedure used to translate the scales. Please be more specific regarding the money participants received. | Thank you for this comment. To address your comment, we have added the following information:  “A bilingual research team translated and back-translated the scales. A university professor in Arabic reviewed the items, and a committee of bilingual researchers finalized the scales. Cognitive interviews with a small group of nurses assessed item clarity.”  When it comes to your second comment about the monetary prizes, we have added this information to address it:  “(two of $25 and one of $50 per hospital).” | Page 10, lines 199-202  Page 10, lines 203-204 |
| R1-19 | In your manuscript, you refer to supplemental material, but I was unable to find it. | We are sorry for this omission. It is now included. | Supplemental material |
| R1-20 | Lines 199-206: who coded implicit power motives and what procedure was followed to do so? | This is now included as supplemental material. | Supplemental material |
| R1-21 | I am not familiar with the measure you used to assess seeking challenges (and was not able to see it as supplemental material was not uploaded). However, to my understanding, the items seem to map also cognitive crafting, which is different than behavioral and does not really align with the dimension of seeking challenges. | Thank you for your comment, the information about the scale is now included as supplemental material.  However, we would like to indicate that the items that we used measure increasing challenging job demands and not cognitive crafting and were adapted from the Tims et al (2012) job crafting scale. So I hope that this addresses your concerns. | Supplemental material |
| R2-4 | 482 nurses from 18 hospitals means a reduced number of participants from each institution. How many nurses answered the survey in each hospital? Please, better inform about this point due to the potential influence of organizational characteristics on job crafting behaviors. | Thank you for your comment. In order to address it, we have added the following information:  “The responses per hospital ranged between 6 and 47 depending on the hospital size. The samples from the different hospitals were similar in gender, age, tenure, and education.” | Page 10, lines 206-207 |
|  | **Results** |  |  |
| R1-22 | It is unclear how moderation was tested. | We tested the moderation by testing the effect of the interaction between implicit and explicit motives while accounting for the direct effect they have on job crafting. We have also indicated that we used Mplus for all our analyses. In order to address your comment, we added the following information: “This indicates that there was a moderation effect.” | Page 15, line 296 |
| R1-23 | The reliability coefficient for Explicit Power Motive is below the commonly accepted threshold. | Thank you for this comment and we try to address this in the supplemental material. Additionally, we have acknowledged this as a limitation in the study. | Supplemental material |
| R2-5 | You have a lot of missing data related to the age. Perhaps, your participants do not want to be identified. Please, explain how anonymity has been guaranteed. | Thank you for your comment and for noticing this! First we would like to indicate that ethical approval was secured from all the institutions of the researchers before collecting the data. Before filling out the survey, the participants had the chance to read about the study and understand how we protect their privacy and anonymity. However, as you have already realized from the demographic information, the majority of our sample were females as the nursing profession is a female dominated one. We also noticed that the participants were hesitant to share their age. We assumed that given that the participants were mostly females, they were less inclined to share their age even after having been reassured that no one will be able to identify them.  We hope that this provided you with more information and reassurance about the data collection procedure that we adopted. |  |
|  | **Discussion and conclusion** |  |  |
| R1-24 | Given the issues with the methods and results (importantly, construct validity and reliability), it is unclear what can be concluded from the study. | We hope that changes that we made and clarifications that we have provided have addressed the issues that your brought up. |  |
| R1-25 | I am not sure if these aspects can be solved with any revision, as they seem pretty fundamental. Should they succeed in doing so, I encourage the authors to rethink their main research question and revise the paper around it, making sure that the value of this investigation is appropriately presented convincingly. | Thank you for your comment. We hope that the changes that we made and the clarifications that we have provided have addressed the issues that you brought up.  In the introduction, we have added more information about the gaps our research addresses. This study delves into the underexplored area of the personal antecedents of job crafting by investigating the involvement of implicit motives in the facilitation of job crafting, which is a novel avenue of inquiry. This investigation is significant, given the argument that job crafting is propelled by basic explicit and implicit motives. Finally, we validate motive congruence in the context of the nursing profession extending our inquiry to encompass a non-western context and concurrently highlighting the positive relationship with work well-being. |  |
| R2-6 | Job crafting behavior is so complex in nurses working environments, as it is a well regulated work-context. Please, discuss this point. | Thank you for this valuable comment. We had addressed it by adding this information:  “Even though the nursing profession is bordered by clinical standard operating procedures, researchers have argued that they can still engage in shape their work within these boundaries (5). This idea has been supported by the numerous studies on job crafting conducted in the context of nursing.” | Page 16, lines 324-327 |
| R2-7 | All the discussion section is too limited. Please, compare your findings with other studies among nurses and with the suggestions and comments of the recent meta-analyses and systematic reviews on Job crafting. | Thank you for your comment. We have added more information in the discussion to try to link our findings to relevant and current findings in a better manner:  “The concept of proactivity and non-compliance with established protocols is not unfamiliar within the nursing discipline, as these can be deliberate efforts to improve current procedures (76)”  “Moreover, a recent meta-analysis by Boehnlein and Baum (81) showed that approach crafting, including increasing challenging job demands, is positively linked to different types of employee well-being including work engagement. We extend this literature by supporting such a robust relationship in an underrepresented non-Western sample and by providing empirical support for the role of motives in increasing the level of motivation among employees (gain spiral) (58).”  “In line with our findings, Bakker and Sanz-Vergel (83)Bakker and Sanz-Vergel (2013) previously showed that when challenging job demands are high, employees with personal resources can thrive and experience higher work engagement. Accordingly, Esteves and Lopes (84) suggested that hospitals provide training for nurses to improve their confidence in taking on challenging job demands.” | Page 16, lines 327-329  Page 18, lines 364-368  Page 19, lines 381- 385 |
