# Supplementary material for: Up for the challenge: Power motive congruence drives nurses to craft their jobs and experience well-being
Source: PLoS One. 2024 Oct 3;19(10):e0310717. doi: 10.1371/journal.pone.0310717 (PMC11449283; doi:10.1371/journal.pone.0310717)
Supplement: Attachment — Submitted filename: Responding to the Reviewers Comments_20240516.docx [file pone.0310717.s005.docx]

Dear Editor, dear reviewers,

We would like to thank you for the valuable feedback that you have provided us with. We believe that this considerably improved our paper, and we hope that the changes that we made fully address your comments.

Below, we have delineated your comments, provided our corresponding responses, and indicated the locations where revisions have been implemented within the paper. We also highlighted the changes that we made in yellow (deleted in red) in the revised manuscript.

We really appreciate the time that you put into revising our paper.

Best regards,

Rawan Ghazzawi on behalf of the co-authors

|  | **Editor’s and Reviewers’ Comments** | **Author’s Response** | **Pages Changed** |
| --- | --- | --- | --- |
|  |  |  |  |
| E1.1 | The revision is appreciated, but not all previous concerns were adequately addressed. | We hope that we have addressed all your concerns with the changes that we have made. |  |
| E1.2 | The justification for focusing solely on seeking challenges in job crafting needs further explanation, as the choice does not seem to align with the strong theoretical support for the relationship between job demands and employee control. | We have added more justification in order to support our choice to focus on increasing challenging job demands. | Lines 99-110 |
| E1.3 | There is a need to clarify why other dimensions of job crafting were overlooked despite the specific request in the previous report. | We hope that the changes that we have made address this comment. | Lines 99-110 |
| E1.4 | Misrepresentation of sources in the manuscript, such as linking explicit power to job crafting without relevant references, raises concerns about theoretical grounding | We have addressed this issue whenever it was pointed out. |  |
| E1.5 | The nested structure of the data should have prompted consideration of multilevel analyses, which appears to have been overlooked. | We appreciate you pointing this out to us, thank you! We provided more information about this in our response below (R1.4) which we hope now addresses this issue. We also conducted an additional analysis to calculate the ICC scores and support of choice to analyze the variables at the individual level. | Lines 288-294 |
| E1.6 | Lack of clarity on how moderation was tested, including the handling of predictors and the impact of adding interaction effects. | We have indicated that we centered the explicit motives variable and not the implicit motives one because the latter is a binary one. Moreover, added some information on how much variance was explained when the moderation was added. | Lines 329-330 |
| E1.7 | Concerns regarding the construct validity of the newly developed scale and its alignment with job crafting behaviors versus motives. | We have addressed this comment by including all the information about the scale development process that we had decided to exclude before for the purpose of conciseness. We hope that this addresses your comment in its entirety, however, if not, we would be happy to elaborate further. | 269-272  And  Supplemental material (S1.a) |
| E1.8 | Uncertainty around how operant motive tests were coded and treated as a continuous variable despite the scale's inherent categorical nature, impacting reliability. | We used it as a binary/ categorical variable as was highlighted in lines 305-306, which is consistent with previous motive research (Baumann & Scheffer, 2010). We hope that this addresses the issue. |  |
| E1.9 | Doubts raised about whether the scale measures job crafting behaviors or motives, emphasizing the need for clarity and validation. | Kindly refer to E1.7 |  |
| E1.10 | Specific item-level critiques, questioning the presence of instrumental motives in certain scale items and the interpretation of performance-related tasks within job crafting | We hope that we addressed the reviewer’s concerns about the specific items in our response to R1.7 |  |
| E1.11 | Issues highlighted in the discussion section regarding the alignment with job crafting concepts, particularly the emphasis on successfully completing challenging tasks versus proactively seeking challenges. | This was adjusted. |  |
| E1.12 | The introduction of the expectancy theory without prior mention or integration into the manuscript underscores the need for a more coherent theoretical framework. | This was removed. |  |
| **R1** | **Introduction** |  |  |
| R1.1 | I do not follow how the fact that the relation between job demands and employee control is strongly supported by the theory justifies your choice of focusing on seeking challenges only (lines 99-100). | We have added more justification in order to support our choice to focus on increasing challenging job demands. | Lines 92-101 |
| R1.2 | Related to this, I’d like to point out that in my previous report, I asked specifically to justify why focusing on seeking challenges only may be appropriate, I never stated you should have considered all job crafting dimensions together. My question was why you only focused on seeking challenges and ignored the other job crafting dimensions. This question remains. | We apologize for not properly addressing your previous question, this was not our intention. We hope that we have now addressed it by the changes that we have made.  We decided to focus on this dimension because it is the one that is most likely to be influenced by this level of power. Taking on challenges at work reflects a level of maturity, which is expressed at the third level of the power motive. Individuals at this level can deal with negative emotions should they arise, which makes them better equipped to tackle challenging job demands because they know that they can finish them. At this level, you do not avoid demands, because you are empowered by positive affect that encourages you to approach tasks at work rather than avoid them. | Lines 92-101 |
| R1.3 | On line 168 you report that job crafting is a planned behavior predicted by high explicit power. However, the references you used to back this statement are not about job crafting at all. | Thank you for pointing this out to us. References (1,5) are about job crafting, however, the references that come after them (48,49) are indeed not about job crafting. However, they were used to explain the motive congruence phenomenon in general. We hope that the changes that we made address you concern. | Lines 166-169 |
|  | **Method:** |  |  |
| R1.4 | It seems that your data has a nested structure, so you should have at least checked whether multilevel analyses are appropriate to test your hypotheses. | We appreciate you pointing this out to us, thank you! We did indeed consider the multilevel, however considering that our data is all at the individual level and indicates individual-level variables (job crafting, implicit and explicit motives, job satisfaction, and work engagement), it was hard for us to use multi-level analysis.  None of the variables that we have collected indicated variability in terms of organizational or team-level behavior. Even if we aggregated the variables to create an organizational-level variable, we would have done that based on no theoretical grounding.  However, we did conduct an additional analysis to calculate the ICC scores and support of choice to analyze the variables at the individual level.  Your comment made us consider this design for the next study, and for that, we will collect data from multiple sources (while making sure we have enough members from each group). | Lines 288-294 |
| R1.5 | When I asked about how moderation was tested, I meant that you should have specified whether you centered your predictors and how the model changed when adding the interaction effect. I know what it means that there is a moderation effect. | Apologies if it came off as us explaining what the moderation effect means, this was not our intention!  We have indicated that we centered the explicit motives variable and not the implicit motives one because the latter is a binary one. I hope this addresses your comment now.  When it comes to the other part of your comment how the model changed when adding the interaction effect, we have now added information about the R-squared before and after including the moderation variables. I hope this addresses your comment, but if not, we are willing to add more information. | Lines 305-306  And  Lines 329-330 |
| R1.6 | Now that the scale is available in the supplemental material, I have more doubts about its construct validity, whether it maps at all job crafting in the form of seeking challenges or whether the constructs that you measure are something very different, both in comparison to job crafting and in the two dimensions proposed, i.e., affective and instrumental motives.  The development of a new scale requires checking whether this relates to the original construct, as well as data to check its validity, which is missing here. Moreover, the procedures used to develop this new scale are rather unclear. I think your scale does not measure job crafting behaviors but rather the motives to engage in job crafting, which is something very different and requires a deeper investigation to understand if this scale you developed is actually valid. | Thank you for your comment, and we agree with you. We have indeed piloted this new scale but decided to exclude this step for simplification and word count. In hindsight, maybe it was not the best decision because it made the process less clear. We have now included this information in the supplemental material. This information shows the different validity tests that we conducted to make sure that we have a valid scale.  When it comes to the other part of your comment, we delineated the original job crafting scale (increasing challenging job demands) by Tims et al. (2012) into instrumental versus effective with an aim to relate it better to explicit and implicit motives. However, as is clear in the pilot study (that is now in the supplemental material) the two sets of items behaved similarly and loaded unto the same major dimensions. Therefore, we ended up using the two set of items as one scale that used the same items as the ones in the original scale by Tims et al (2012), but that added a motivational element to them. We also reported the CFA of ONE job crafting scale instead of two, which shows that we have used it as one scale in the analysis. Based on your comment, and in order to avoid any confusion, we made this clear under the job crafting section under measures and we hope that the information about the validation process that we added also makes it clearer.  When it comes to your comment about the scale itself, and while we do see your point (measuring motives instead of behaviors), we think that it still measures actual behavior. We think so for two main reasons: the first is the fact that we adapted these items from the original scale that measures JC behavior by Tims et al (2012), while not diverting a lot in terms of wording. We just kept the behavior and added the motivational element to it. The second reason is a more technical one as this adapted JC scale behaved in a way similar to the original scale (positively related to proactive personality and personal initiative and negatively related to cynicism).  We hope that this addresses your comment in its entirety, however, if not, we would be happy to elaborate further. | 269-272  And  Supplemental material (S1.a) |
| R1.7 | As another issue, (so please, in case you are invited to resubmit a revised version, I’d appreciate a response to each of my comments), while in most of the items, I can somehow see how instrumental considerations are expressed, I do not see this in the item “I try to understand how my work tasks are related to one another”. Where is the instrumental motive here? Similarly, I found it unclear whether the item “I take on extra tasks because this is part of my performance appraisal” actually maps some instrumental motive that comes from within the employee rather than as something that is expected – I would assume that if something is part of your performance appraisal you don’t really have much choice about whether to do it or not. | We see your point when it comes to this item. However, before we provide you with our reasoning, we would like to point out that we tried our best to stay as close as possible to the original scale. This meant that we did not change the items that, based on face value, looked instrumental. Having mentioned that, our reasoning for adapting this item in such a way was to highlight that employees engage in job crafting in order to “understand” how their work tasks are related to one another so that they can work better instead of enjoying this process, which we thought would be more affective.  When it comes to the second item that you have highlighted, and again just as the case with a lot of points that you bring forth, we see what you mean and we agree with it. However, we think that taking extra tasks because you will avoid punishments/ receive benefits can be considered instrumental. This was also our way to make sure that there is a clear difference between what you are doing because you have to versus because you enjoy it.  We hope that this addresses your comment in its entirety, however, if not, we would be happy to elaborate further |  |
| R1.8 | Similarly to the scale developed to measure affective and instrumental job crafting motives, I have some serious concerns regarding the way you coded operant motive tests. From what I read in the supplement file, it seems that you treated it as a continuous variable, but then the scores from 1 to 4 refer to approach crafting and 5 refers to avoidance crafting, i.e., something completely different. Yet, you treat it all together as a single continuous variable. This is a serious concern, and I think can partially explain the issues with the unacceptable reliability. | In the supplemental file, we described how coders differentiate the five levels of motive enactment. Even if aggregating scores across all five levels is the classical way to measure the general power motive, we were specifically interested in the integrative power motive (level 3) in this study. Thus, we used only level 3, that is, the integrative motive enactment. Nevertheless, you raise an excellent point and this would have been an issue had we considered all five levels together as a continuous variable. However, we used only level 3 as a binary/ categorical variable as was highlighted in lines 311-312. We hope that this addresses the issue. |  |
| R1.9 | In the discussion, you start by saying that successfully completing challenging job demands is important. Yet, job crafting is not about that, it’s about proactively looking for more challenging job demands. | Thank you for pointing this out to us; we agree with you. We have changed the discussion section. | Lines 357-358 |
| R1.10 | On line 406 you cite the expectancy theory of motivation, but this comes across as rather out of the blue, given that it wasn’t mentioned in the intro nor anywhere in the manuscript. This further highlights the lack of a coherent theoretical framework underlying this study. | We see your point and accordingly, we have removed the expectancy theory of motivation to avoid the impression of lacking coherence. | Lines 414-419  Highlighted in red |
| R1.11 | Line 411-413 – I do not understand how reference 83 is relevant to your study findings. | We have included this study because we thought that it is in line with the findings of our study: employees who increase their challenging job demands and who have high personal resources (which is motive congruence in our case) experience higher levels of work engagement. We have added another sentence that articulates how we think our study links to the Bakker and Sanz-Vergel one. | Lines 419-423 |
| R1.12 | The theoretical contributions are not well grounded in the existing literature. It is unclear how they specifically advance what we already know. | Thank you for your comment. We hope that the changes that we made have addressed your comment properly. | Lines 432-437 |
| **R2** |  |  |  |
| R2.1 | In the introduction it will be more advised to include information about the nature & current situation of job crafting in healthcare settings in Lebanon (how common is it among researchers in the same region), which is usually governed by ministries or authorities at national level. And in order to bridge the gap and implement the recommendations, policy makers (specifically human resources managers) need to be convinced about it. | Thank you for your comment! While we do understand the value of adding more context information, we think that, just like studies conducted in the Western part of the world, we should not always make our studies (that are conducted in the non-Western part of the world) about the context only. Context does matter and this is why we alluded to it in different parts of the paper, however, it is not within the scope of the paper. We are investigating universal individual differences in a very standardized profession, which allows us to assume some generalizability. However, as we mentioned first, we see a lot of value in your comment, and this is why the practical note that will be based on the published paper, will provide more information about the context of Lebanon for policy maker. |  |
| R2.2 | Usually, this topic will be combined by assessment of burnout, which was missing, using Maslach Burnout Inventory as example. The MBI had a section about personal achievement that can be considered as part of positive outcomes of job crafting. | Thank you for this comment, we agree with you, however, this was beyond the scope of the study and we felt that the topic of burnout among nurses has received a lot of attention in the literature so far. We were worried that including this variable would not have addressed major gaps in the literature. Notwithstanding, we have added this suggestion to the limitations section. | Lines 481-484 |
| R2.3 | Line 69, a contradicting statement (implicit motives require objective techniques because they are not consciously accessible to individuals) | Thank you for pointing this out, it was a typo! | Line 62 |
| R2.4 | Lack comparison with other professions, in reference to the nature of healthcare settings and presence of multidisciplinary approach. | Thank you, we have added information about this throughout the paper. We hope that this addresses your comment. | Lines 130-131  Lines 362-364 |
