# Supplementary material for: Up for the challenge: Power motive congruence drives nurses to craft their jobs and experience well-being
Source: PLoS One. 2024 Oct 3;19(10):e0310717. doi: 10.1371/journal.pone.0310717 (PMC11449283; doi:10.1371/journal.pone.0310717)
Supplement: Attachment — Submitted filename: Responding to Editor s Comments_20240816.docx [file pone.0310717.s006.docx]

| Dear Editor,  We would like to thank you for the valuable feedback that you have provided us with. We believe that this considerably improved our paper, and we hope that the changes that we made fully address your comments.  Below, we have delineated your comments, provided our corresponding responses, and indicated the locations where revisions have been implemented within the paper. We also highlighted the changes that we made in yellow (deleted in red) in the revised manuscript.  We really appreciate the time that you put into revising our paper.  Best regards,  Rawan Ghazzawi on behalf of the co-authors | | | |
| --- | --- | --- | --- |
|  | **Editor’s Comments** | **Author’s Response** | **Line number** |
| **1.1** | The abstract needs to be shortened and made more accessible to a wider audience. | Thank you for this comment. We shortened the abstract and filtered out the terms that were too technical and could be mentioned at a later stage throughout the manuscript. | Lines 24-37 |
| **1.2** | It should clarify the difference between needs and self-attributed needs, and explain how power motives fit within the context of job crafting, which typically relates to the self. | This was not mentioned in the abstract, however, we made sure to clarify the difference the first time we mentioned these terms in the manuscript. | Line 119 |
| **1.3** | Additionally, the study design should be better described without specifying all hypotheses. | We added more information about the study design and removed the hypotheses. | Lines 28-36 |
| **1.4** | Terms like “integrative power motive enactment” are unclear and need explanation, particularly the focus on seeking challenges. | This term was not used throughout the paper, however, we added information in the text that we hope makes this link clearer. | Lines 139-142 |
| **1.5** | It is also essential to justify why increasing challenges is considered a critical dimension of job crafting. | We have added information in the abstract that we hope addresses this comment. | Lines 31-32 |
| **2.1** | The introduction should provide a more comprehensive review of the job crafting literature, acknowledging that previous research has addressed basic motives such as power, affiliation, and achievement.. | Given that the research on the link between job crafting and basic needs/motives is very limited, we made sure to include all of the studies that have addressed this relationship. However, we think that adding more information can indeed strengthen our introduction and accordingly we have added more details in the second paragraph of the introduction that we hope addresses your comment. | Lines 57-60 |
| **2.2** | Unnecessary details, such as correlation coefficients, should be removed. | We have removed this information. | Line 104 |
| **2.3** | A stronger rationale for the study is needed, focusing on the main research question and its importance. | We agree with you that the main research question was not clearly indicated, and accordingly, we have added one we hope makes the main research aim of the study clearer. | Lines 77-79 |
| **2.4** | The description of job crafting should accurately distinguish between increasing challenging demands and decreasing hindering demands. | We have added more information about the difference between increasing challenging job demands and decreasing hindering job demands. | Lines 93-98 |
| **2.5** | References and variance reports must be accurate, and. | Thank you for indicating that, we have reviewed the paper to make sure that this information is accurate. | Throughout the paper. |
| **2.6** | the argument that nursing is driven primarily by power motives should be reconsidered, exploring other potential motives | We hope that the changes that we have made make this argument more convincing. | Line 136 |
| **2.7** | Recent research on job crafting behaviors and cognitive crafting should be included | Thank you for this suggestion, however, we think that cognitive crafting is beyond the scope of our study and adding it will add even more concepts and distract the reader. | No change. |
| **2.8** | the extensive use of acronyms should be reduced for better clarity and consistency | Thank you for this comment, however, the previous versions of the paper did include a lot of acronyms and based on the comments that we received, we reduced them. Based on your comment, we reviewed the paper again and made sure there are no unnecessary acronyms. | Throughout the paper. |
| **3.1** | Methodology: Details on participant recruitment, the information provided regarding study aims, and translation procedures for scales need to be specified. | We have added more information about participant recruitment under the sample and procedure section. In the current version of the study, we have included information about how the scales were translated “Questionnaires were administered in Arabic after careful scale adaptation processes [72, 73]. A bilingual research team translated and back-translated the scales. A university professor in Arabic reviewed the items, and a committee of bilingual researchers finalized the scales. Cognitive interviews with a small group of nurses assessed item clarity.”  We hope that this information addresses your comment. | Lines 228-229 |
| **3.2** | Participant compensation details should also be included. | We have added that participants were not compensated for participating in the study. | Lines 236-237 |
| **3.3** | It is crucial to ensure that all supplemental material is accessible. The process and personnel involved in coding implicit power motives should be clearly outlined. | We have indeed provided information about the coding process in the supplemental material: “Coding the answers was conducted by the first author who is bilingual (English, Arabic), and underwent coding training with the second author until the percentage of agreement between the trainer and the trainee reached more than 80%.”  We hope that this information addresses your comment. | Supplemental material S1.b. |
| **3.4** | The measure used to assess seeking challenges should align with behavioral job crafting rather than cognitive crafting. | We are glad that you think so because this is exactly what we aim to measure. Cognitive crafting is beyond the scope of your study and was not measured as part of the research that we have conducted. | No change. |
| **4.1** | Results and Analysis: The moderation testing process requires clarification. | We have added information about the variables that we included in the moderation analysis. We hope that this makes the moderation analysis clearer. | Lines 339-340 |
| **4.2** | The reliability of the Explicit Power Motive measure is below the commonly accepted threshold and needs to be addressed. | This is very true and accordingly, we have included a section in the supplemental material that addresses this issue. We also refer to this section in the paper. | Supplemental Material S1.c. |
| **4.3** | Additionally, the number of statistical tests should be reduced to maintain clarity and impact. | Thank you for this suggestion, however, we do not know which statistical tests we should remove without impacting the transparency of our analysis. We would appreciate it if you can provide us with the specific tests that you want to be removed so that we can move them to the supplemental material. | No change. |
| **5.1** | Discussion and Conclusion: Given the issues with methods and results, particularly regarding construct validity and reliability, the study's conclusions are unclear | We hope that the changes that we made based on your comments have addressed these issues. We really appreciate your help and support throughout this process and hope that the current version of the paper has clearer conclusions. | Throughout the paper. |
| **5.2** | Concerns about the originality of the research, ambiguity in findings, and limited generalizability need to be addressed. | When it comes to the originality of the research, this study is the first to link implicit motives to job crafting, which makes it very original. Based on your comment, we have strengthened the research gap mentioned in the introduction, so we hope that this addresses your concern. Moreover, we have included limitations related to the findings and the generalizability of the study under the “Limitations and Future Research Directions” section that we hope addresses your concerns. | Lines 475-502 |
